# Fish Scales Produce Cortisol upon Stimulation with ACTH

**DOI:** 10.3390/ani12243510

**Published:** 2022-12-12

**Authors:** Athanasios Samaras, Michail Pavlidis

**Affiliations:** Department of Biology, University of Crete, Voutes University Campus, 70013 Heraklion, Greece

**Keywords:** adrenocorticotropic hormone, cortisol, chronic stress, in-vitro stimulation, fish, scales

## Abstract

**Simple Summary:**

Cortisol is the most commonly studied acute stress indicator in fish. Recently, it has been shown that cortisol can be measured in fish scales, and its concentration can reliably indicate a response to chronic stress. The mechanism of the increase of cortisol concentration in scales has been proposed to be accumulation through circulation; however, no study has so far examined whether the scales can produce and release cortisol. Therefore, in the present study the ability of isolated fish scales to produce and secrete cortisol after incubation with cortisol-stimulating ACTH was examined. Results show that ACTH administration increased cortisol release in a dose-dependent manner, an effect that was reversed when scales were incubated with an inhibitor of cortisol production, i.e., metyrapone. This is the first study to report peripheral cortisol producing abilities in fish scales.

**Abstract:**

Cortisol concentration in fish scales is a novel and reliable indicator of chronic stress. However, until now cortisol in scales has been considered to be accumulated through the circulation and it has not yet been studied whether it can be de novo produced from cells found in the scales. In the current study, scales of European sea bass, *Dicentrarchus labrax*, were stimulated in-vitro with a range of concentrations of adrenocorticotropic hormone (ACTH) to investigate if they can produce and release cortisol. Moreover, scales were exposed to a combination of ACTH and metyrapone, an inhibitor of cortisol production, to examine whether cortisol was actually produced in the scales. Results from ACTH administration showed that scales increased their cortisol release in a dose-dependent manner. This effect was reversed when scales were co-incubated with ACTH and metyrapone, indicating that cortisol was produced de novo and not released only upon stimulation with ACTH.

## 1. Introduction

Fish in both natural and cultured conditions are commonly faced with noxious stimuli and stressors. In general, circulating blood cortisol is considered to be one of the most reliable and commonly used indicators of acute stress [1,2], though under chronic stress it has been reported that it is not able to reliably indicate the stress load that fish have been exposed to [3,4,5,6]. For that reason, alternative indicators of chronic stress evaluation are needed, and methods for measuring cortisol concentration in matrices other than plasma have been developed and validated during the last few years [7]. Among these indicators, cortisol concentration in the scales of fish has been reported to be the most reliable in assessing chronic stress [4,6,8,9]. Specifically, Aerts et al. [6] reported that while plasma cortisol showed no differences between control and chronically stressed carp, *Cyprinus carpio*, the concentration of cortisol in the scales was significantly increased in the latter. The same result was observed in rainbow trout, *Oncorhynchus mykiss*, by another research group [8], and has also been verified recently by our research group in European sea bass, *Dicentrarchus labrax* [4].

Cortisol in fish scales, similar to corticosterone in bird feathers [10] and cortisol in mammalian hair [11], is considered to accumulate through the circulation of blood, and therefore it has been proposed to depict periods of elevated plasma cortisol levels due to exposure to chronic stress [6]. However, in mammals it has been shown that cortisol is not only accumulated in hair via the circulation, but it is also locally produced in the skin or hair follicles [12,13]. Specifically, in mammals it has been well established that the skin [14,15], and in more detail melanocytes [16], fibroblasts [17], as well as hair follicles [18], produce cortisol through the local synthesis of all products of the classical Hypothalamus–Pituitary–Adrenal (HPA) axis [19]. CRH, urocortins, ACTH, a-MSH and b-endorphin, cortisol, as well as their respective receptors, namely CRHR1 and 2, melanocortin receptors, and glucocorticoid receptors have been reported to be expressed and produced in fish skin [19].

In fish it has not yet been investigated whether cells in the skin, the scales or other associated tissues and organs can produce cortisol. Fish scales are skeletal elements deriving from the dermis. They are located in the skin and are covered by the epidermis at their posterior end, while the anterior part is sunk into the dermal stroma [20]. Although cycloid and ctenoid fish scales primarily contain type I collagen fibers and minerals, mainly hydroxyapatite, cells are also present. Common cell types found in the scales or attached to them include osteoclasts and scleroblasts [21], as well as pigment cells, such as melanocytes and to a lesser extent xanthophores, of epidermal origin [22,23,24].

In this context, the aim of the current study was to examine whether fish scales detached from the fish can produce cortisol when stimulated with ACTH. To do so, isolated scales of European sea bass were cultured in-vitro and stimulated with different concentrations of ACTH. Moreover, to determine whether cortisol was produced de novo, European sea bass scales were additionally incubated in medium containing metyrapone, which is an inhibitor of cortisol production. Finally, scales were also simultaneously cultured with ACTH and metyrapone to test whether the ACTH-induced release of cortisol would be reversed due to the inhibitory action of metyrapone on cortisol production or not.

## 2. Materials and Methods

### 2.1. Incubation Protocol

Scales were collected from sexually immature European sea bass fish (mean weight ± SD: 128.1 ± 10.5 g) reared in 500 L open-flow tanks, at a temperature of 19 °C, a salinity of 38, and a dissolved oxygen saturation between 95–100%. The collection of scales was performed using forceps to carefully remove the scales. Due to the fact that a large number of scales was needed (200–300 mg per fish), fish were euthanized with a high dose of 2-phenoxyethanol (500 ppm; Merck 80729, Burlington, MA, USA) immediately before the collection of scales. The collected scales were placed in an in-vitro culture system consisting of 96-well plates and incubated at 19 °C (to coincide with the rearing temperature of the fish) in 15 mM HEPES/Tris buffer (pH 7.4) culture medium containing 171 mM NaCl, 2 mM KCl, 2 mM CaCl2, 0.25% (*w*/*v*) glucose, 0.03% (w/v) bovine serum albumin and 0.1 mM ascorbic acid.

Before placing in a 96-well plate, scales were rinsed with culture medium to wash away the mucus. Afterwards, 40–60 mg of scales was incubated in 200 ul of culture medium for 2 h in order for cortisol release to reach an equilibrium (the time interval was selected based on preliminary experiments using fish scales combined with data from [25] concerning superfusion of European sea bass head kidneys). The first experiment was performed to investigate whether ACTH administration could stimulate the release of cortisol from scales. For that reason, scales from each fish were divided into five experimental groups and incubated for 1 h in 200 ul of culture medium containing ACTH_1-39_ (Sigma-Aldrich, A0423, Germany) in the following concentrations: (1) 10^−6^ M ACTH; (2) 10^−7^ M ACTH; (3) 10^−9^ M ACTH; (4) 10^−11^ M ACTH; and (5) no ACTH (control). After 1 h, the medium was collected for cortisol analysis and stored at −20 °C until analysis. During this experiment, head kidney and liver samples were also collected and incubated under the same experimental conditions to test the efficacy of the methodology to study changes in cortisol production. Head kidney is the site of cortisol production in fish, and therefore incubation with ACTH should lead to the production and release of ACTH in the culture medium (positive control) [25], whereas the liver is not capable of producing cortisol, and therefore no cortisol production and release should be observed after ACTH administration (negative control).

A second experiment was performed, using the same procedure, to test the effects of metyrapone on cortisol production. For this experiment, scales were divided into six experimental groups and incubated with the following concentrations of metyrapone (Sigma-Aldrich, 856525, Taufkirchen, Germany) with and without ACTH: (1) 10^−4^ M metyrapone; (2) 10^−6^ M metyrapone; (3) 10^−4^ M metyrapone + 10^−6^ M ACTH; (4) 10^−6^ M metyrapone + 10^−6^ M ACTH; and (5) no metyrapone or ACTH (control) (6) 10^−6^ M ACTH (positive control). The medium was collected as previously described.

The first experiment was independently repeated three times and the second experiment two times, both using four fish in each repetition. Cortisol concentration in the incubation medium was quantified using commercially available ELISA assay (Neogen Corporation, Ayr, UK) in duplicates. The medium was measured directly without prior extraction after being tested and showing no cross-reactivity with the incubation medium. The intra- and inter- assay variation of the assay were 9.7% (±2.8%) and 15,8% (±4.8%), respectively, while the parallelism was 0.961(*n* = 5).

### 2.2. MTT Assay

To examine tissue viability during the incubation, the Thiazolyl Blue Tetrazolium Bromide (MTT) assay was performed on whole scales as previously described for animal tissues [26,27], with a few modifications in order to perform the assay on a 96-well plate. In brief, after the incubation described above was completed, 10–15 mg of scales from a subset of samples were incubated for 2 h in 96-well plates in 200 ul MTT (2 mg ml^−1^; Sigma-Aldrich, M2128, Taufkirchen, Germany) dissolved in the incubation medium on a rotating platform. Subsequently, scales were removed from the well, rinsed with incubation medium to discard any MTT remnants and minced with scissors. Formazan was then extracted from the minced scales in 300 ul of DMSO, agitating in a rotating platform. The absorbance of formazan was read at 540 nm, and results were calculated as abs/mg of tissue and expressed as % tissue viability compared to control samples that were treated with MTT immediately after the dissection without being cultured in the above-mentioned experimental set-up. Only samples stimulated with the highest concentrations of ACTH, metyrapone or the combination of metyrapone and ACTH were analyzed. As a negative control, samples incubated in DMSO were used to obtain the lowest possible absorbance.

### 2.3. Statistical Analysis

Statistical analysis was performed using GraphPad Prism 7.0 (GraphPad Software, San Diego, CA, USA). Before the analysis, data were tested for normality using the Kolmogorov-Smirnov and the Shapiro-Wilk tests, and for equality of variances using the Brown-Forsythe test. In the experiment of cortisol release after ACTH stimulation normality and equality of variances assumptions were violated, and log-transformation was applied on the data since the assumptions were met following this transformation. One-way ANOVA followed by Tukey’s post-hoc tests was performed on cortisol release data at a significance level of *p* < 0.05.

## 3. Results

### 3.1. Cell Viability under In Vitro Culture of the Scales

**Figure 1:**
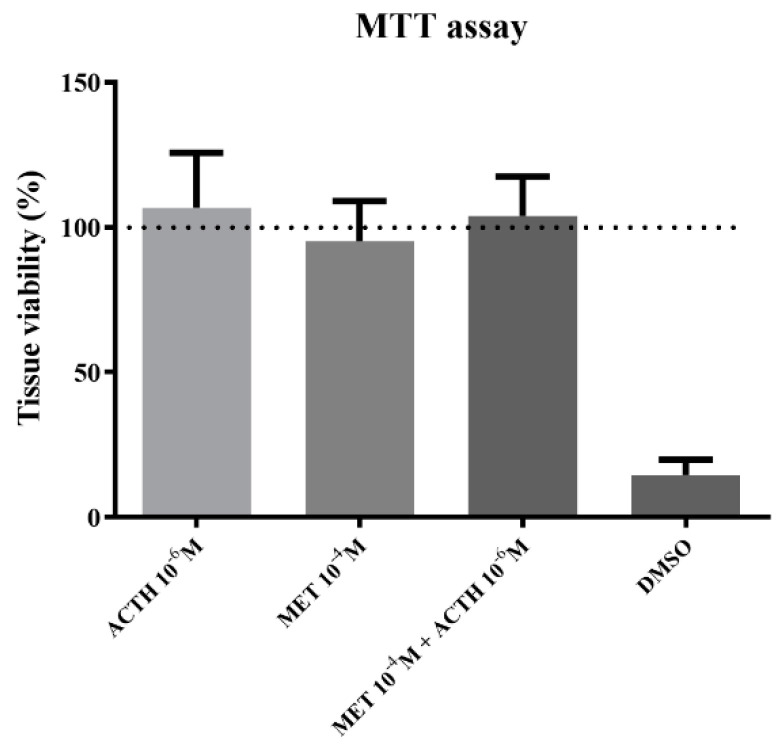
Tissue viability compared to control (expressed as %) after 1 h incubation with 10^−6^ M ACTH; 10^−4^ M metyrapone; simultaneous incubation with 10^−6^ M ACTH and 10^−4^ M metyrapone; DMSO. Results are expressed as average + SD.

Cell viability was not affected due to incubation with the highest concentrations of ACTH, metyrapone or the combination of those two (Figure 1). On the other hand, incubation with DMSO led to cell death and therefore reduced cell viability compared to control.

The set-up for scale incubation allowed the detection of cortisol release from scales to the incubation medium. The average (±SD) cortisol release in the control samples was 0.22 (±0.21) pg mg^−1^ h^−1^, with a minimum value of 0.04 and maximum value of 0.78 pg mg^−1^ h^−1^. Additionally, in order to test the accuracy of the incubation method, head kidney and liver samples were used as positive, and negative controls, respectively. Incubation of head kidneys with 10^−6^ M and 10^−7^ M ACTH led to a 4.8 (±0.9) and 2.1 (±0.4) fold-increase compared to controls, respectively (*n* = 4). In contrast, liver showed no stimulation of cortisol release when stimulated with 10^−6^ M ACTH (*n* = 4).

### 3.2. Cortisol Release after ACTH Administration

**Figure 2:**
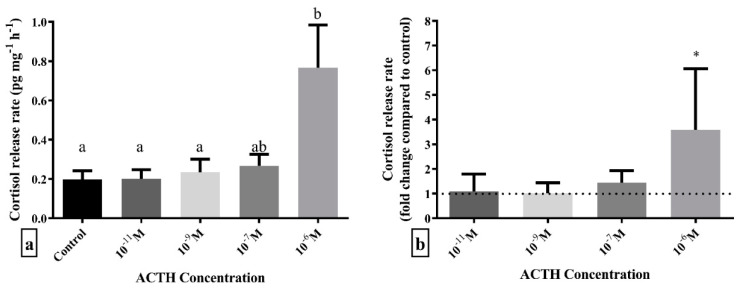
Cortisol release rate expressed as (**a**) pg cortisol per mg of scales per hour, pg mg^−1^ h^−1^ and (**b**) fold-change compared to control (dashed line) after 1 h in vitro incubation with different doses of ACTH. Results are expressed as average + SD (number of individuals in each trial *n* = 4, number of trials *N* = 3). Different letters indicate statistically significant differences between groups. Asterisks indicate statistically significant fold-change differences compared to control.

Incubation with different doses of ACTH showed that only the highest dose of 10^−6^ M led to a statistically significant higher release of cortisol from scales compared to control (F_4,55_ = 3.438; *p* = 0.014) (Figure 2). Post hoc analysis revealed that scales incubated with 10^−6^ M ACTH had statistically significant higher cortisol release rate than those incubated without ACTH, as well as 10^−9^ M and 10^−11^ M ACTH, while 10^−7^ M ACTH was between these groups.

### 3.3. Cortisol Release after Metyrapone Administration

**Figure 3:**
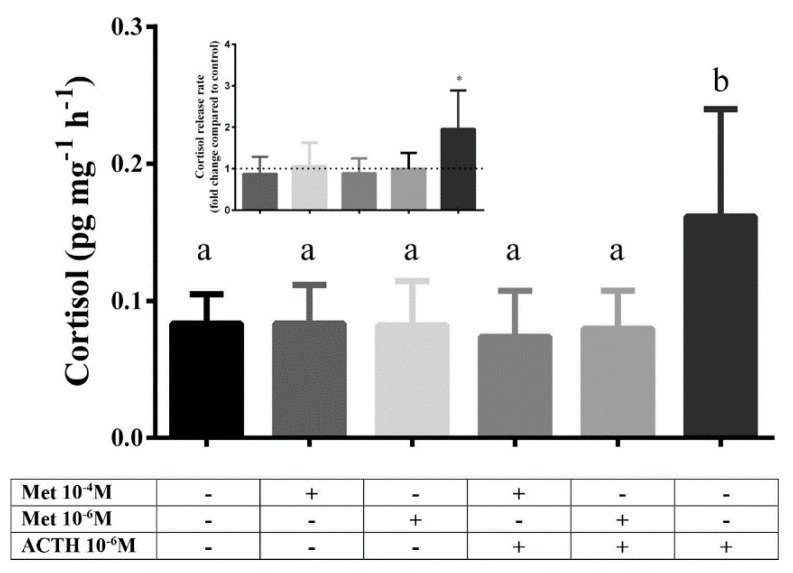
Cortisol release rate (pg cortisol per mg of scales per hour, pg mg^−1^ h^−1^) after 1 h in vitro incubation with different doses of metyrapone, ACTH and combination of metyrapone with ACTH. Results are expressed as average + SD (number of individuals in each trial *n* = 4, number of trials *N* = 2). Different letters indicate statistically significant differences between groups. Inlet: cortisol release rate expressed as fold change compared to control (dashed line). Asterisks indicate statistically significant differences compared to control.

In the second experiment a statistically significant difference between groups was observed (F_5,42_ = 3.179; *p* = 0.016). Post hoc analysis showed that only the group stimulated with ACTH 10^−6^ M, as a positive control, was different compared to all other groups. Therefore, incubation with metyrapone had no effect on cortisol release rate compared to control, regardless of the tested dose. Combined incubation with metyrapone (10^−4^ M or 10^−6^ M) and ACTH (10^−6^ M) inhibited the ACTH-derived stimulation of cortisol release (Figure 3).

## 4. Discussion

The in-vitro release of cortisol from fish scales was studied for the first time, and showed that the administration of ACTH was able to stimulate it. Moreover, incubation with metyrapone, a substance that blocks cortisol biosynthesis by acting as a reversible inhibitor of 11-beta hydroxylase, abolished ACTH-derived cortisol release from scales. These data offer support for the notion that scales detached from the body as previously described do not only accumulate, but also produce cortisol upon stimulation with ACTH. However, the results do not indicate the biological components of the fish scales that produce cortisol, since scales consist of a matrix consisting mainly of collagen fibers and mineralized layers, as well as various cells types such as osteoblasts, scleroblasts and melanophores, the latter most probably a remnant of the epidermis covering the scale [21].

Until now, the elevation of cortisol concentration in fish scales due to chronic stress had been considered a result of cortisol accumulation through blood circulation [6,8], and the biosynthetic capacity of the scales had not been studied. Fish scales are of dermal origin, initiating from the dermis and projecting upwards towards the epidermis, and they comprise the dermal skeleton of the fish. In mammals, peripheral cortisol production in skin tissues, such as the hair follicles, and cells found in the skin such as melanocytes and fibroblasts, have been reported, and a mechanism for the production and secretion of cortisol has been described [16,17,18,19]. Moreover, in mammals there is evidence for a local and peripheral expression and action of all the elements of the HPA axis, including CRH, ACTH, cortisol and their respective receptors [16,17,18,19].

Metyrapone, a substance that blocks synthesis of cortisol from 11-deoxycortisol led to the abolishment of ACTH-stimulated increase in cortisol release from the samples. This indicates that cortisol was synthesized de novo following ACTH administration, and not merely released. Although this is the first time that metyrapone has been used in a study examining cortisol release from scales, results from in vivo experiments in live animals [28,29,30] and in vitro experiments using head kidney tissue in fish [29] report that metyrapone is a potent inhibitor of post-stress cortisol production. However, under basal conditions, it seems that both a single administration [31] or multiple treatments of metyrapone [32] were not able to reduce basal cortisol levels, which were only reduced when fish were treated orally, via food, with multiple administrations of high dose metyrapone [32]. These results are in accordance with the lack of reduction in cortisol in the metyrapone-treated samples observed in the present study.

Regarding the overall mechanism of cortisol production from the fish integumentary system, including the scales, epidermis, dermis and skin appendages, it remains unclear if the same mechanisms function in fish as in mammals, but the current results provide the first indication towards that direction. We did not study the mechanisms of cortisol production, and to the best of our knowledge all the studies that have examined the expression of genes involved in cortisol in the integumentary system have been performed in skin tissue. Specifically, it is already known that European sea bass skin tissue expresses the receptor of ACTH, i.e., MC2R, [33]. This underlines the fact that in sea bass skin the mechanism of reception of the ACTH signal is present, since MC2R has ACTH as an exclusive ligand [33]. Moreover, after signaling from ACTH, cortisol is produced from cholesterol by a series of reactions catalyzed by the action of a series of steroidogenic enzymes, of which steroidogenic regulatory protein (StAR) and 11-beta hydroxylase (Cyp11b1) are crucial [2,34,35]. However, it remains unclear whether fish express steroidogenic enzymes in their scales or skin. Specifically, although no data for European sea bass are available in the literature, the expression of genes encoding for StAR [36] and 11-beta dehydrogenase (HSD11B) [37,38] have been documented in zebrafish, *Danio rerio*, and Atlantic salmon, *Salmo salar*, skin, respectively, which could explain the inhibitory action of metyrapone on ACTH-stimulated cortisol production observed in the current study. On the other hand, in rainbow trout, no expression of StAR [39] and 11-beta hydroxylase (Cyp11b1) [40] genes have been observed in their skin, suggesting a possible species-specificity in the regulation of skin cortisol dynamics, underlying the necessity for further investigation towards the mechanisms of function of cortisol production in fish skin and scales.

## 5. Conclusions

Taken together, the results of the current work point out that scales detached from the fish skin, and carrying layers epidermal cells apart from their own cells, produced and released cortisol after incubation with ACTH, a function that was reversed when they were co-incubated with ACTH and metyrapone, an inhibitor of steroidogenesis. Although the mechanisms of action of cortisol production were not studied, the current study is the first to show that peripheral tissues, specifically detached scales, can produce cortisol; a finding that can serve as a starting point for further research in that direction.

## Data Availability

Data available upon reasonable request.
